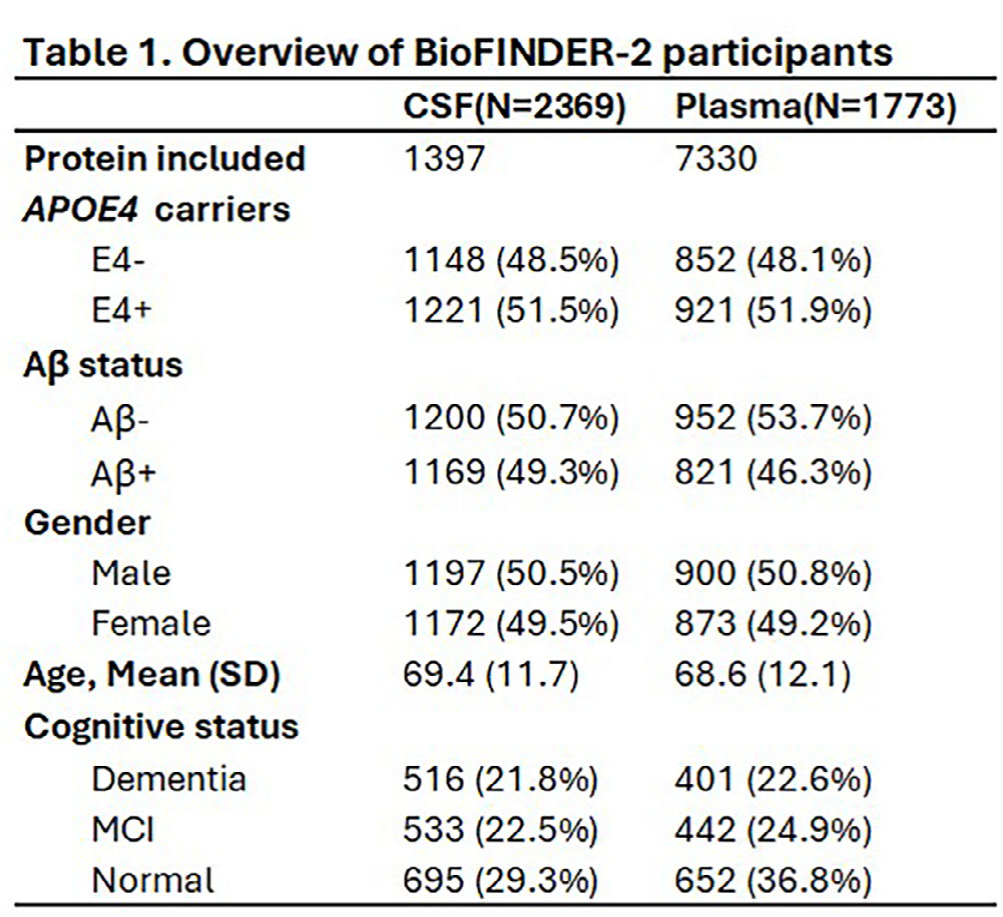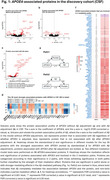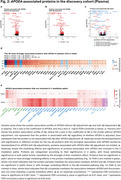# *APOE ε4*‐related proteomic profiles in plasma and CSF

**DOI:** 10.1002/alz70856_103395

**Published:** 2025-12-26

**Authors:** Lina Lu, Alexa Pichet Binette, Shorena Janelidze, Erik Stomrud, Jacob W. Vogel, Sebastian Palmqvist, Rik Ossenkoppele, Oskar Hansson, Niklas Mattsson‐Carlgren

**Affiliations:** ^1^ Clinical Memory Research Unit, Department of Clinical Sciences Malmö, Faculty of Medicine, Lund University, Lund, Sweden; ^2^ Department of Clinical Sciences Malmö, SciLifeLab, Lund University, Lund, Sweden; ^3^ Clinical Memory Research Unit, Lund University, Malmö, Skåne, Sweden

## Abstract

**Background:**

The *APOE ε4* allele (*APOE4*) is a major genetic risk factor for Alzheimer's disease (AD) and is strongly linked to amyloid‐beta (Aβ) pathology. However, the mechanisms by which *APOE4* influences Aβ remain unclear. This study aims to identify proteins related to *APOE4* via both Aβ‐independent and dependent pathways using proteomics.

**Method:**

We included 1,773 participants with plasma proteomics (SomaLogic 7K) and 2,369 participants with CSF proteomics (Olink Explore3072) from the BioFINDER‐2 cohort. We examined proteins associated with *APOE4* (carrier vs. non‐carrier) or Aβ status (based on CSF Aβ42/Aβ40), independently and after adjusting for each other. Mediation models were applied to identify *APOE4*‐associated proteins mediating the effects of *APOE4* on Aβ (Figure 1e, Path1: *APOE4* = > protein => Aβ) and those mediated by Aβ (Figure 1e, Path2: *APOE4* = > Aβ => protein). All analyses utilized linear models, adjusted for age, sex, and mean protein levels.

**Result:**

In CSF, 98 out of 1,397 proteins were associated with *APOE4*. Of these, 9 *APOE4*‐associated proteins (e.g. SNAP25) were not affected by adjusting Aβ (Figure 1d). Aβ fully or partially mediated the effects of *APOE4* on 76 proteins (e.g. SMOC1), with the mediation proportion ranging from ‐43% to 179% (Figure 1f). In CSF, most *APOE4*‐protein associations were attenuated after Aβ adjustment while most Aβ‐protein associations remained independent of *APOE4* (Figure 1a,b,c). Conversely, in plasma, *APOE4*'s effect on most proteins remained even after Aβ adjustment (Figure 2a,b). In plasma, 254 out of 7,330 proteins were associated with *APOE4*, 172 proteins were not affected by adjusting Aβ (Figure 2d). 8 proteins partially mediated the effect of *APOE4* on Aβ (e.g. SPC25), ranging from 1% to 28%, while Aβ fully or partially mediated the effect of *APOE4* on 29 proteins, ranging from ‐27% to 66% (Figure 2e). Investigation of possible differences across platforms and cohorts are ongoing.

**Conclusion:**

Our findings reveal distinct proteomic profiles associated with *APOE4* in plasma and CSF. Complex molecular mechanisms are linked to *APOE4*, both with and without the development of AD pathology. Proteins implicated in *APOE4*‐related pathways may be explored as potential biomarkers for early detection and therapeutic targets.